# Supplementary figures and images for: Mediating Effects of Trait Anxiety and State Anxiety on the Effects of Physical Activity on Depressive Symptoms
Source: Int J Environ Res Public Health. 2023 Mar 30;20(7):5319. doi: 10.3390/ijerph20075319 (PMC10094143; doi:10.3390/ijerph20075319)

Supplementary Figure S1.  
Age<60 group

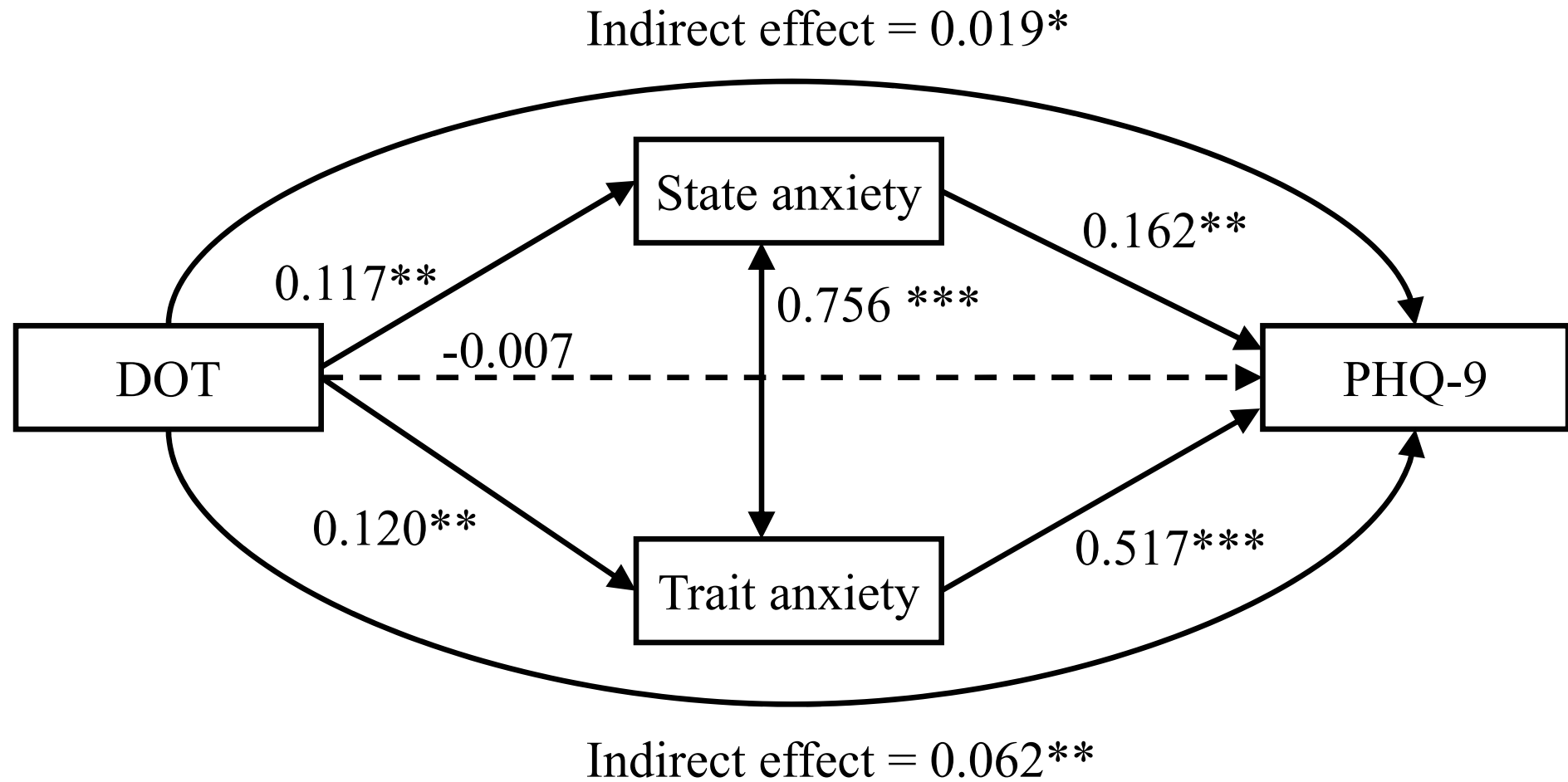

Supplementary Figure S2.  
Age $\geq$ 60 group

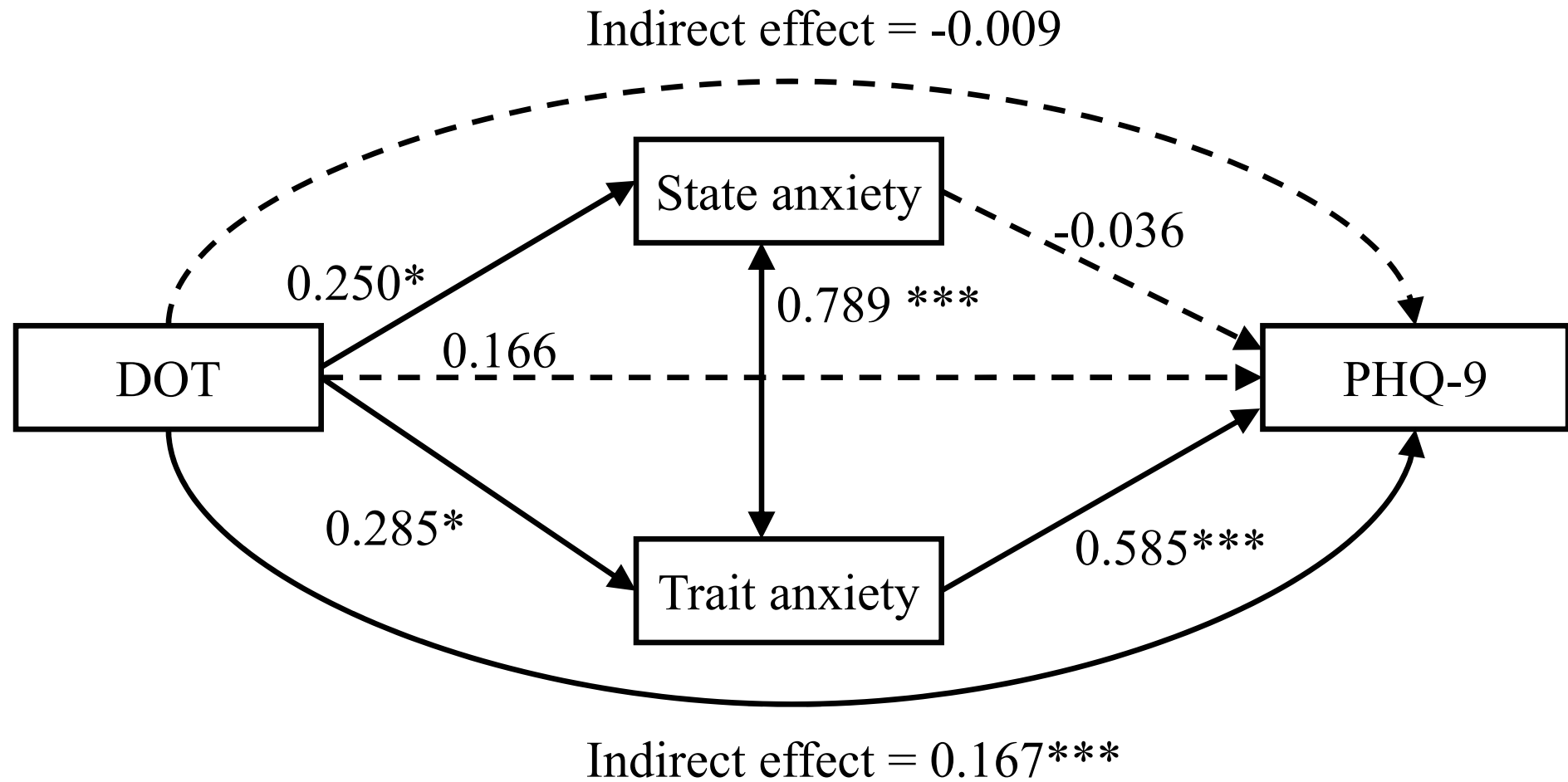

Supplement: Supplementary file 1 [file ijerph-20-05319-s001.zip › Supplementary Figures S1 and S2.pdf]
